# Differential Expression of Genes that Control Respiration Contribute to Thermal Adaptation in Redband Trout (*Oncorhynchus mykiss gairdneri*)

**DOI:** 10.1093/gbe/evv078

**Published:** 2015-05-04

**Authors:** Michael R. Garvin, Gary H. Thorgaard, Shawn R. Narum

**Affiliations:** ^1^School of Biological Sciences, Washington State University; ^2^Columbia River Inter-Tribal Fish Commission, Hagerman, Idaho

**Keywords:** respiration, environmental genomic interaction, positive selection, mitochondria, complex I, hypoxia

## Abstract

Organisms can adapt to local environmental conditions as a plastic response or become adapted through natural selection on genetic variation. The ability to adapt to increased water temperatures will be of paramount importance for many fish species as the climate continues to warm and water resources become limited. Because increased water temperatures will reduce the dissolved oxygen available for fish, we hypothesized that adaptation to low oxygen environments would involve improved respiration through oxidative phosphorylation (OXPHOS). To test this hypothesis, we subjected individuals from two ecologically divergent populations of inland (redband) rainbow trout (*Oncorhynchus mykiss gairdneri*) with historically different temperature regimes (desert and montane) and their F1 progeny to diel cycles of temperature stress and then examined gene expression data for 80 nuclear- and mitochondrial-encoded OXPHOS subunits that participate in respiration. Of the 80 transcripts, 7 showed ≥ 2-fold difference in expression levels in gill tissue from desert fish under heat stress whereas the montane fish had none and the F1 only had one differentially expressed gene. A structural analysis of the proteins encoded by those genes suggests that the response could coordinate the formation of supercomplexes and oligomers. Supercomplexes may increase the efficiency of respiration because complexes I, III, and IV are brought into close proximity and oligomerization of complex V alters the macrostructure of mitochondria to improve respiration. Significant differences in gene expression patterns in response to heat stress in a common environment indicate that the response was not due to plasticity but had a genetic basis.

## Introduction

As the climate continues to change, adaptation to environments with fluctuating temperature regimes will become important for many at-risk species. Adaptation may be possible in the form of phenotypic plasticity that is not rooted in molecular evolution but is rather a physiological response to changes in environmental conditions (West-Eberhard 2003; Ghalambor et al. 2007; Chevin et al. 2010; Beldade et al*.* 2011). However, adaptation may also occur through natural selection on genetic variation that renders individuals within a deme more “fit” in certain environmental regimes (henceforth referred to as “adaptive or adaptation”). If this is the case, it may be possible to identify genes that are important for this process of adaptive evolution.

The rainbow trout (*Oncorhynchus mykiss*) is a widely distributed species that has acclimatized to diverse environments in the wild as well as conditions that are optimal for production and aquaculture; they have been transplanted so that they are now globally distributed (MacCrimmon 1971). One source of environmental variation across their native and nonnative environments is water temperature. The optimal growth temperature for this species is approximately 14 °C and yet populations of redband trout (a subspecies of rainbow trout in the Columbia River Basin, *O. mykiss gairdneri*) have been observed in desert streams with temperatures that reach nearly 30 °C (Rodnick et al. 2004; Zoellick 1999). An increase in water temperature reduces dissolved oxygen; according to Henry’s Law, fresh water at 30 °C will have 20% less oxygen than at 15 °C. Thus, climate change is expected to cause oxygen to become limited for aquatic organisms and their adaptive response will be critical for persistence in local environments (Pörtner and Knust 2007). Salmonid fishes from differing climates have been shown to have evolved enhanced cardiac function to persist in environments with higher water temperatures and lower oxygen availability (Eliason et al. 2011). Previous studies of desert and montane ecotypes of redband trout have also demonstrated differences in survival under heat stress and confirmed patterns of evolutionary adaptation to thermal conditions (Narum et al*.* 2010, 2013).

**Fig. 1.—:**
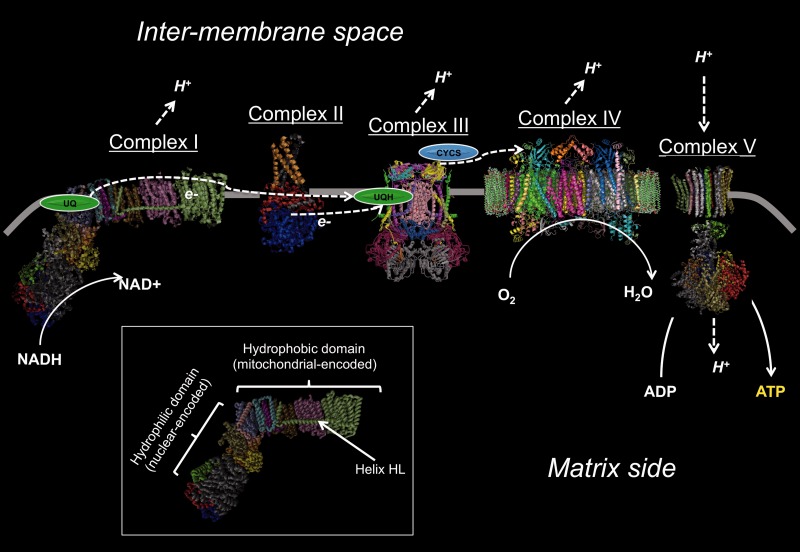
Schematic of OXPHOS. Complex I oxidizes NADH to NAD+. The electrons from that reaction are carried from complex I to III through ubiquinone (UQH) and from complex III to IV through cytochrome *c* (CYCS) where they bind with oxygen to form water. Complexes I, III, and IV form the proton gradient that complex V uses to generate ATP from ADP. The helix HL that was shown to be a target of diversifying selection is shown (inset).

In all animals, respiration occurs in the mitochondria with five complexes (complexes I–V) through oxidative phosphorylation (OXPHOS; Lodish et al*.* 2000). Complexes I, III, and IV generate a proton gradient that is driven by the oxidation of NADH (fig. 1). The electrochemical potential that is generated by the movement of protons is subsequently used by complex V to generate ATP from ADP. In parallel to the proton movement, the electrons that were produced from the oxidation of NADH are transported through complexes I and III and then bind oxygen to generate water at complex IV.

There are 13 mitochondrial genes in metazoans that encode subunits for four of the five complexes, and it has been well established that positive diversifying selection acts on these proteins as part of local adaption and speciation in diverse taxa (da Fonseca et al*.* 2008; Garvin et al*.* 2015). More than 70 nuclear-encoded subunits of these complexes appear to coevolve with the mitochondrial-encoded subunits (Gershoni et al. 2010; Garvin et al. 2011, 2015; Puslednik et al. 2012; Parmakelis et al. 2013; Zhang and Broughton 2013) and it has also been recently shown that complexes I, III, and IV form higher order structures called “supercomplexes” (Dudkina et al. 2011; Lapuente-Brun et al. 2013) that enhance respiration, possibly by decreasing the distances among active sites (Kadenbach 2012) or improving the stability of complexes (Blaza et al. 2014).

As metabolic pathways have been shown to be differentially regulated in redband trout populations under heat stress (Narum and Campbell 2015), this study specifically tested OXPHOS genes to investigate their contribution to local adaptation of redband trout from extreme temperature regimes where altered respiration would be necessary. Fish from ecologically divergent populations (desert, montane, F1 hybrids) were tested for differential gene expression across 80 OXPHOS genes under heat stress in a common laboratory environment. Both gene-expression and sequence data were examined from each treatment group for 80 OXPHOS genes to identify differences among populations. If a respiratory response were due to simple phenotypic plasticity, we would expect similar patterns of gene expression from all groups whereas differences among groups in an experiment with common environmental conditions would indicate evidence for evolutionary adaptation.

## Materials and Methods

### Study Design

To test for expression differences in OXPHOS genes in ecologically divergent populations of redband trout (*O. mykiss gairdneri*), gametes and fry were collected from a desert and montane population located in Idaho, USA as described in previous studies (Narum et al. 2013; Narum and Campbell 2015). Briefly, the desert stream was Little Jacks Creek (LJ, from here forward referred to as “desert”) and the montane stream was Keithly Creek (K, from here forward referred to as “montane”), and K females were crossed with LJ males in order to create F1 crosses (from here forward referred to as “hybrids”). The two strains have spawn times that differ by approximately 1 month so it was not possible to create reciprocal hybrids in this first generation of fish captured from wild populations. In order to create the single direction F1 cross (K × LJ), milt was cryopreserved from the earlier spawning desert strain (LJ) and used to fertilize eggs from montane fish (K) about 4–5 weeks later. Fry of approximately 4 months of age from the desert and montane ecosystems and their F1 crosses were exposed to diel temperature cycles over a 4-week period in a controlled setting. Diel water temperatures representing summer conditions from the desert stream ranged from 17°C overnight and peaked at approximately 28°C for 1 h, with temperature changes of about 1.5°C per hour (Narum et al. 2013). Each of these three strains was also reared for the same duration in control tanks at a constant 15°C. Gill tissues were collected from euthanized individuals on days 1, 3, 7, and 28 from both control (15°C) and treatment (28°C) tanks to quantify mRNA expression. All experimental protocols were approved in advance by the University of Idaho’s Institutional Animal Care and Use Committee (Protocol #201025).

Libraries for RNA sequencing (RNA-Seq) were prepared as described in Narum and Campbell (2015) and run on an Illumina HiSeq1500. Briefly, total RNA was normalized to 100 ng/µl and equal volumes of RNA from three fish from each tank were pooled for a total of 72 libraries (3 strains × 2 temperatures × 3 replicate tanks each × 4 time periods each). Pooled RNA samples were taken through three steps during library preparation following manufacturer protocols for 1) Ribo-Zero Magnetic Gold Kit (Epicentre) to remove ribosomal RNA, 2) strand-specific library preparation with ScriptSeq v2 RNA-Seq Kit (Epicentre, Madison, WI), and 3) ScriptSeq (Epicentre) to add index polymerase chain reaction primers. Each of the pooled libraries was sequenced in two lanes of a single read 100-bp flow cell on an Illumina HiSeq 1500 instrument for a total of 16 lanes. Reads per sample after quality filtering ranged from 22.40 to 72.40 M per sample with an overall mean of 31.96 M.

### Sequences

For this study, the archived Illumina RNA-Seq files were downloaded from the SRA (Sequence Read Archive) database (entry GSE53907) and converted to FASTQ format with the SRA Toolkit available on the National Center for Biotechnology Information (NCBI) website. The putative RNA-editing and single nucleotide polymorphism (SNP) variants were identified with a pipeline of the RNA-editing/Mutation algorithm in the T-bioinfo platform (http://tauber-bioinfo.haifa.ac.il, last accessed May 18, 2015). Briefly, the 72 next-generation sequencing samples were mapped onto the mRNA sequences of 80 genes of interest. For each sample, a putative variant at a position was found as a mutation with the highest count and with the significant variant calling score. This score was estimated from the confidence interval for the position-specific binomial distribution that is based on the smallest count variant at the position.

In order to investigate differences in gene expression, FASTQ files were uploaded to CLC Genomics Workbench for analysis. The mRNA coding sequences for each of 76 known genes (62 nuclear- and 13 mitochondrial-encoded subunits of complexes I–V as well as the electron carrier cytochrome *c*; supplementary table S1, Supplementary Material online) were determined for the desert and montane populations by first creating a local Basic Local Alignment Search Tool (BLAST) database for each population. We then performed a BLAST search for sequences for the 76 genes for *O. mykiss* that we extracted from GenBank to obtain sequences that were specific to each population to identify any fixed differences between populations in the coding region of each gene. In the cases of *COX6B, COX8B, COX6C*, and *QCR6B*, we identified what at first appeared to be fixed differences but were then classified as potential isoforms, which were then confirmed with a search of the EST (expressed sequence tag) database at NCBI for all four genes. These were added to the list of genes for which we obtained RNA-Seq data for a total of 80 mRNA sequences (supplementary table S1, Supplementary Material online). There were no fixed sequence differences between the desert and montane populations in the coding region of any of the OXPHOS genes.

We then used the RNA-Seq function in CLC Genomics Workbench to extract the transcript counts for each of the 80 genes. The mRNA counts from nuclear- and mitochondrial-encoded subunits were normalized separately because the amounts can differ in a sample. Each animal cell contains a single nucleus with a diploid copy of nuclear DNA, but cells can vary widely in the numbers of mitochondria per cell and the copy number of mitochondrial DNA per mitochondrion. Recall from above that three fish were pooled into each sample and therefore each SRA file represents information from three combined individuals. We normalized each nuclear gene from each SRA file by dividing by the sum of all of the nuclear-encoded transcript counts for that pool of three. This was done separately for the mitochondrial-encoded genes. These measures in essence represent the proportion of the total mRNA for each of the genes in each sample. We multiplied each expression index value by 1,000 to facilitate mathematical manipulations.

### Statistical Analyses

We performed a two-way analysis of variance for the response of each gene and the predictor variables temperature (15 or 28 °C) and population (desert, montane, or hybrid). The normalized count data were first log-transformed to reduce false positives that can result from outliers. For the response to temperature, values were averaged across all time points and for comparisons among populations, the transcriptome counts from the 15 °C group were averaged across time points. Post hoc tests were performed to determine significance within nested groups. We corrected for multiple tests with a false discovery rate (BY-FDR) of 0.01 (Narum 2006). For genes that were determined to be significantly different between two groups, we reported the fold-difference of the normalized mRNA counts. In order to determine basal levels of gene expression, we first tested for differential expression among strains under control temperatures at 15 °C with the ratio of desert/montane, desert/hybrid, and montane/hybrid. The mean transcript count at all four time points was used.

### SNP/RNA Editing Detection

Chi-square statistics were used to test for the significance of each particular SNP across samples of a population (LJ, K, or F1); across samples, sum of squares of scores of each SNP were measured in confidence interval units. A significant difference between populations was performed with an *F*-test based on a ratio of two chi-square statistics.

### Structural Analysis

Protein Data Bank (PDB) files 2YBB (supercomplex of I, III, and IV), 4HEA (complex I), 1PPJ (complex III), 1V54 (complex IV), and 1E79 with 1C17 (complex V) were rendered and colored with the Visual Molecular Dynamics viewer 1.9.1 (Humphrey et al. 1996). For the supercomplex, we show views that are rotated 90° from each other.

## Results 

No fixed differences between desert and montane samples were identified in a survey of 80 nuclear- and mitochondrial-encoded genes. We also looked for different numbers of polymorphic sites among the three groups and found nine SNPs that appeared in both LJ and K but not in the F1 hybrid fish (table 1). Seven of the nine SNPs were located in mitochondrial-encoded subunits, which may reflect loss of mitochondrial haplotypes during the generation of the hybrids. Only one resulted in a nonsynonymous change (*QCR7*, glycine to glutamic acid). Five of the nine SNPs were A to G transitions and may reflect RNA editing. We were unable to accurately estimate allele frequencies for these sites among our samples because our RNA was pooled, but future studies may be able to address this.

**Table 1:** SNPs Found in the Little Jacks and Keithly Samples but not in the F1 Hybrids

Gene Position	Mutation
QCR07‐116^a^	G > A
ATP5O‐537	**A > G**
MTCO2‐624	C > T
MTCO3‐324	C > A
MTCYTB‐945	A > T
MTCO1‐1056	**A > G**
MTCO1‐1281	**A > G**
MTCO2‐594	**A > G**
MTCO3‐459	**A > G**

Note.—Bold letters indicate those that are potential sites of RNA-editing.

^a^Nonsynonymous.

Below we report the gene expression data as fold-change for the genes that were differentially regulated at a statistically significant level. Differential regulation is presented in two cases. The first is the difference between strains at control temperatures (15 °C) to establish base levels of gene expression and the second is differences between control (15 °C) and treatment (28 °C) for each of the three groups (desert, montane, and hybrids). Subsequent to the presentation of these data we give a brief summary of the function of each complex. Complex II is not presented because the two genes in our data set were not differentially regulated at the statistical level in any comparison. Finally, we discuss the four isoforms that we found and their significance.

### Differences among Strains at Control Temperatures (15 °C)

Our comparison of the gene expression levels among the three groups at 15 °C revealed only three genes that were differentially regulated and all were from the mitochondrial genome. The gene for *ND3* was lower (−1.3, *P* > 0.01) in the desert population compared with montane and the hybrids, *CYTB* (cytochrome b) was higher (+1.2, *P* < 0.001) in the hybrids compared with montane, and *CO1* was higher (2.0, *P* < 0.01) in the desert compared with the hybrids.

### Differences among Strains under Heat Stress (15 vs. 28 °C)

#### Complex I—NADH:Ubiquinone Oxidoreductase

We obtained sequence and gene-expression information for 38 mitochondrial- and nuclear-encoded subunits for complex I (supplementary table S1, Supplementary Material online, and table 2). The mRNA transcripts for the mitochondrial-encoded *ND1* and *ND5* subunits were upregulated (*P* < 0.01) in the desert fish in response to increased temperature as were three (*NDUFS2, NDUFV1*, and *NDUFV2*, *P* < 0.01, *P* < 0.001, and *P* < 0.01, respectively) of the seven nuclear-encoded subunits of the hydrophilic core (the remaining four are *NDUFS1, NDUFS3, NDUFS7*, and *NDUFS8*). The nuclear-encoded supernumerary subunits *NDUFB8* and *NDUFS6* (*P* < 0.001) were also upregulated in the samples from the desert population. The *NDUFS6* subunit was recently reported to interact with *NDUFS1, NDUFS2*, and *NDUFS8* (Vinothkumar et al. 2014).

**Table 2:** Summary of Significantly Expressed Genes among Strains

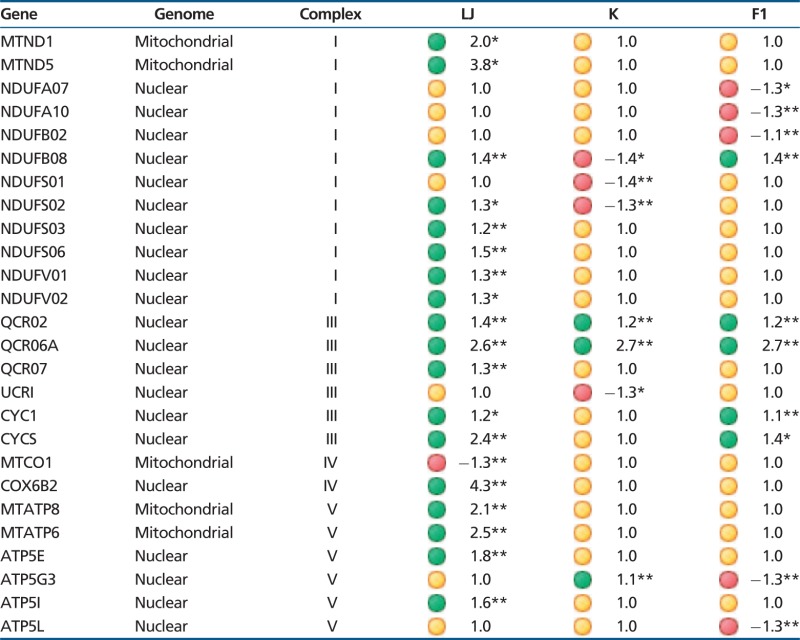

Note.—Numbers indicate fold difference between the two treatments. Green dots represent significantly upregulated genes, red significantly downregulated, and yellow not significantly different among groups.

* *P* < 0.01; **P < 0.001.

In contrast, *NDUFA7, NDUFA10*, and *NDUFB2* were downregulated in the hybrid fish in response to an increase in temperature (*P* < 0.01, *P* < 0.001, *P* < , 0.01, respectively). *NDUFA10* is located above the ND2 subunit on the matrix side of the inner membrane and phosphorylation of this subunit has been shown to be necessary for complex I activity (Morais et al. 2014; Vinothkumar et al. 2014). The gene for NDUFB8 was upregulated in the hybrids similar to desert population (*P* < 0.001 each), but was downregulated in the montane fish (*P* < 0.01). Finally, *NDUFS1* and *NDUFS2* were both downregulated in montane individuals (*P* < 0.001) but not in the hybrids nor the desert fish.

Mitochondrial complex I is the first and the largest enzyme in the chain of complexes that participate in OXPHOS (fig. 1). The seven nuclear encoded-subunits of the “core complex” reside in the hydrophilic domain and participate in the oxidation of NADH and transfer of electrons to ubiquinone. The membrane-bound hydrophobic domain consists of the four main proton “pumps” that are formed by seven highly conserved mitochondrial-encoded ND subunits. The 30 + supernumerary subunits are not necessary for the catalytic activity (Hirst 2011), but can regulate it and some of their locations are now known (Vinothkumar et al. 2014).

The transfer of two electrons to ubiquinone is accompanied by the movement of four protons across the membrane, which contributes about 40% of the total proton flux to generate the electrochemical gradient for ATP synthesis. Proton translocation appears to be driven by conserved charged amino acids that are located along the central axis that traverses the four proton pumps (Baradaran et al. 2013; Hirst 2013; Zickermann et al. 2015). The movement is likely also coordinated by a long lateral helix HL that acts as a “coupling rod” (Efremov and Sazanov 2011) in the C-terminal portion of ND5 (although see Vinothkumar et al. 2014).

#### Complex III—Ubiquinol:Cytochrome c Oxidoreductase

In complex III, we found that the genes for *QCR2* and *QCR6A* were upregulated in response to temperature in all three populations of fish (*P* < 0.001; table 2). The electron carrier *cytochrome c* (CYCS) and *CYC1* were upregulated in the desert (*P* < 0.01, *P* < 0.001, respectively) and hybrid fish (*P* < 0.001, *P* < 0.01, respectively). CYC1 transfers electrons from one of the iron-sulfur proteins of complex III to cytochrome *c. QCR2* is involved in the formation of the homodimer for this complex and the gene for QCR6A has been shown to interact with cytochrome *c* (Nakai et al. 1993). *QCR7* was upregulated in the desert fish in response to temperature (*P* < 0.001) and the gene for *UCR1* was downregulated in this strain (*P* < 0.01).

The main reaction of complex III is the “*Q*-cycle” (Trumpower 1990). In the first phase of the cycle, as two electrons are removed from the ubiquinol that was shuttled from complex I, the complex releases two protons into the inner membrane space that contribute to the electrochemical potential (fig. 1). One of the electrons is transferred to soluble cytochrome *c*, which continues on to complex IV. The second electron is transferred to ubiquinone to form a semiquinone. In the second part of the cycle, another ubiquinol binds cytochrome *b*, two electrons are again removed and two protons are again transferred across the inner membrane. As before, one of the electrons is transferred to cytochrome *c*, which continues on to complex IV. In the second phase, an electron is ultimately transferred to the semiquinone, and with the addition of two protons, ubiquinol is regenerated—completing the cycle. High-resolution structures reveal ten nuclear-encoded subunits surrounding the core cytochrome *b* subunit that is central to the transfer of electrons and concomitant proton movement of this complex (Xia et al. 1997; Iwata et al. 1998; Gao et al. 2003).

#### Complex IV—Cytochrome c Oxidase

In complex IV, the mRNA transcript for *COX6B2* is significantly upregulated (*P* < 0.001) in the desert strain and that of the mitochondrial-encoded gene *CO1* is downregulated in this strain (*P* < 0.001; table 2). Complex IV transfers the electrons from cytochrome *c* that were passed from complex III to oxygen to form water (Tsukihara et al. 1996; Yoshikawa et al. 1998; Kim and Hummer 2012). As with complexes I and III, electron transfer is linked to proton translocation to contribute to the proton gradient.

#### Complex V—ATP Synthase

The mRNA for the two mitochondrial-encoded subunits of complex V (*MTATP6* and *MTATP8*) and two nuclear-encoded subunits (ATP5E and ATP5I) were upregulated (*P* < 0.001; table 2) in the desert fish in response to temperature but not in the samples from montane or hybrid fish. A single gene (*ATP5G3*) was upregulated in montane but downregulated in hybrid individuals in response to an increase in temperature (*P* < 0.001). Finally, the gene for *ATP5L* was downregulated in the hybrids but not the parental strains (*P* < 0.001). The subunits for ATP5L, ATP5I, MTATP6, and MTATP8 have all been shown to be necessary for the creation of oligomers for the ATPase complexes.

Complex V comprised a catalytic hydrophilic (F_1_) portion and a hydrophobic (F_0_) structure that forms the proton channel in the membrane. A high-resolution structure for the F_1_ portion has been reported (Watt et al*.* 2010) and the mechanism of action has been deduced (Deckers-Hebestreit and Altendorf 1996; Dickson et al. 2006). A high-resolution structure for the F_0_ section, in which the mitochondrial-encoded subunits (ATP6 and ATP8) reside, has yet to be determined but their likely placement and role in the formation of oligomers has been reported (Rak et al. 2011; Jonckheere et al. 2012; Habersetzer et al. 2013).

#### Isoforms

Isoforms represent variants of a gene that can perform the same function, a different function altogether, or a slight modification of the same function (Little et al. 2010). They can exist as the result of different transcription start sites, splice variants, or as paralogs that arose from whole-genome duplication events (as is the case in salmonids) (Christensen et al. 2013) followed by divergence through mutation (in the absence of positive or negative selection they often become nonfunctional pseudogenes). It appears that the COX isoforms all arose through gene duplication and the regulation of complex IV activity occurs in-part through the expression of different isoforms of the nuclear-encoded subunits in different tissues and in response to different physiological conditions (for an extensive review, see Arnold 2012b).

The most studied isoforms of cytochrome *c* oxidase are the subunits for COX4; one isoform is typically expressed ubiquitously in humans (COX4-1) and the other in fetal tissue, adult lung, and neuronal cells (COX4-2; Vijayasarathy et al. 1998; Arnold 2012a, 2012b). These isoforms are differentially expressed in response to hypoxia and toxins in cells lines (Arnold 2012b) as well as exercise in humans (Desplanches et al. 2014). Mice that have had the isoform for *COX4-2* knocked out display reduced complex IV activity and respiratory ability (Huttemann et al. 2012).

The isoforms for *COX6*, *COX7*, and *COX8* are typically described as a “heart” and “lung” isoforms due to their tissue-specific expression. The “heart” holoenzyme appears to have a lower proton/electron stoichiometric ratio compared with the “lung” form (Frank and Kadenbach 1996), which supports the idea of differential expression in response to differing respiratory needs. In mammals, one of the isoforms for COX6 is testes-specific and appears to be in response to the high aerobic capacity of that cell type (Huttemann et al. 2003).

Our BLAST search to identify population-specific sequences for redband trout identified isoforms for *COX6B* (*B1* and *B2*), *COX6C* (*C1* and *C2*), *COX7* (*B* and *C*), and *COX8* (*B1* and *B2*). Of these, *COX6B2* was expressed at low levels overall but was upregulated in the desert fish in response to temperature (table 2). We also identified isoforms for subunit QCR6 (QCR6A and QCR6B) that are part of complex III and that to our knowledge have not been described before. The QCR6A isoform was upregulated in response to increases in temperature in all fish but the 6B isoform was not.

#### Gene Expression by Genome

The desert fish from LJ demonstrated the largest number of differentially expressed genes for both the nuclear (20.3%) and mitochondrial (38.5%) genomes compared with the montane and hybrid fish (table 3). The hybrid fish were intermediate between the parental strains at the nuclear genome, and matched that of the montane fish (their female parent) for the mitochondrial genome.

**Table 3:** Summary of the Number of OXPHOS Genes that Are Differentially Expressed between Control and Treatment Conditions by Genome Type and by Group

Complex	Genome	LJ	K	F1
CI	Nuclear	6	3	4
CI	Mitochondrial	2	0	0
CII	Nuclear	0	0	0
CII	Mitochondrial	N/A	N/A	N/A
CIII	Nuclear	5	3	4
CIII	Mitochondrial	0	0	0
CIV	Nuclear	1	0	0
CIV	Mitochondrial	1	0	0
CV	Nuclear	2	1	2
CV	Mitochondrial	2	0	0
% Nuclear		20.3	10.1	14.5
% Mitochondrial		38.5	0.0	0.0

Note.—Each cell refers to the number of statistically significant differentially expressed genes out of 80 targets examined.

## Discussion

Our results indicate that the respiratory response of redband trout has likely evolved to become locally adapted in ecologically divergent populations of this species and cannot solely be attributed to phenotypic plasticity. Expression profiles for several genes that participate in OXPHOS are statistically significantly different among populations of redband trout from desert and montane habitats when subjected to heat stress in a common laboratory environment. Our previous study in these strains demonstrated decreased mortality in montane fish in response to short-term heat exposure due to high expression of heat shock proteins, but desert-adapted fish had lower mortality under long-term heat exposure (Narum et al. 2013). Furthermore, the desert fish from LJ demonstrate numerous genes throughout the transcriptome that are differentially regulated in response to increases in water temperature that are not seen in the montane fish from K (Narum and Campbell 2015). This suggests that fish from desert climates have evolved capabilities to tolerate warm water temperatures and low oxygen content whereas montane fish have limited response capabilities due to lack of molecular adaptation under these environmental conditions.

The OXPHOS genes may be the target of adaptive evolution or a compensatory response to the targeted molecular network. Regardless, it appears that montane fish may lack adequate molecular machinery or possibly decrease respiration in response to heat as is suggested by the fact that almost all of the differentially regulated OXPHOS genes are downregulated. Downregulated genes included *NDUFA10*, which regulates complex I activity through phosphorylation by another protein (Morais et al. 2014). Notably, most of the changes in the desert fish involve upregulation and not downregulation and they include genes that encode proteins from four of the five OXPHOS complexes. This suggests that the response in desert fish involves the coordination of the entire respiratory machinery. Furthermore, functional roles of genes that were differentially regulated imply that regulation involves reorganization of complexes at the ultrastructural level.

**Fig. 2.—:**
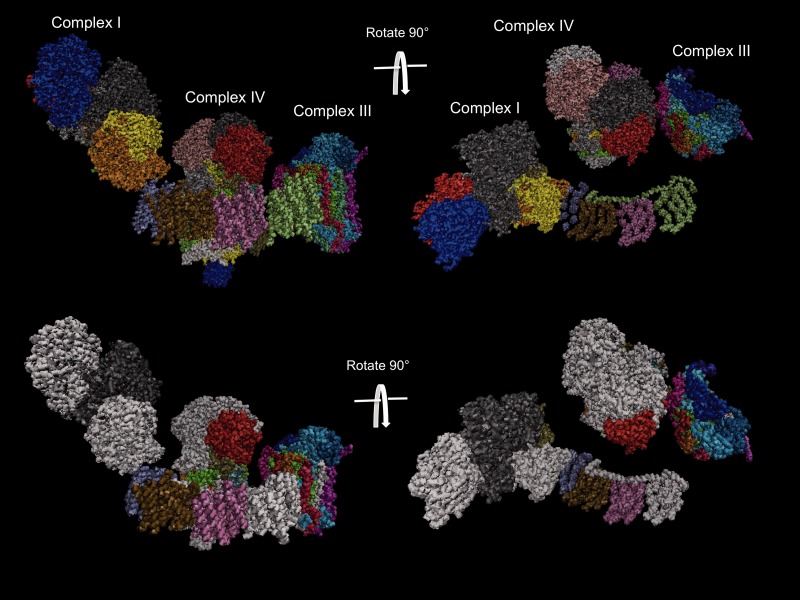
A three-dimensional rendering of the supercomplex formed by complexes I, III, and IV. Each subunit is represented by a different color in each complex (top). Subunits that are differentially regulated in the desert strain as part of the response to increased temperature are shown in white (bottom).

The *COX6B2* isoform was upregulated in the desert fish in response to temperature. COX6B lies at the interface between monomers of complex IV and is one of the subunits that initiates the formation of the active, functional homodimer (Pierron et al. 2012). Different isoforms of nuclear-encoded COX subunits have been shown in many species to be important for the regulation of respiration and confer different activities on complex IV in different tissues and under different oxygen concentrations (Arnold 2012a; Pierron et al. 2012). It is possible that switching from the 6B1 to the 6B2 isoform here in gill tissue alters the formation of the homodimer, increases the stability of the complex itself or stabilizes interactions with other complexes, that is, supercomplexes (fig. 2) (Dudkina et al. 2008).

The gene for *QCR7* from complex III is also upregulated in the desert fish in response to increased temperature. Like COX6B, this protein subunit lies at the interface between the two halves of the homodimers of the complex in which it resides and therefore could also increase or decrease the stability of the complex itself. Additionally, QCR7 has been shown to interact with COX6B in order to form a supercomplex between complexes III and IV (Zara et al*.* 2007). Complex I forms supercomplexes with the active homodimers of complexes III and IV that appear to interact with the face of complex I through the helix HL connecting arm (Dudkina et al. 2011). This connecting arm is encoded by the ND5 protein that is upregulated in the desert fish and the addition of this protein during complex I assembly was recently shown to promote supercomplex formation (Vartak et al. 2015). These results suggest that adaptation to higher water temperature in fish from LJ involves increased expression of genes that are regulatory points for the formation of higher-order supercomplexes.

Three of the five genes for complex V that are upregulated in the desert population in response to temperature are necessary for the formation of oligomers. The formation of oligomers has been shown to influence the ultrastructural organization of mitochondria and the internal cristae at the macroscopic level; the inability to do so decreases the respiratory capacity from OXPHOS (Habersetzer et al. 2013). Like the upregulation of genes for complexes I, III, and IV, the genes for subunits of complex V also appear to affect the ultrastructural organization of the respiratory machinery. In summary, the identity and location of the differentially expressed subunits imply that the functional result of the gene expression patterns is to alter interactions among monomers within complexes and interactions among complexes.

Without high-resolution structures for salmonids it is difficult to determine whether changes in isoforms increase or decrease these interactions but increases in complex superstructure stability have been shown to increase the efficiency of respiration, perhaps by bringing subunits in closer proximity to increase the efficiency of the transfer of intermediates (Lenaz and Genova 2012). However, carefully controlled molecular physiology experiments determined that physical channeling among subunits is unlikely (Blaza et al. 2014). The authors of that work suggest that supercomplex formation and oligomerization of complex V is a means to increase the number of functional respiratory units within a mitochondrion while preventing aggregation. A reanalysis of the respiratory capacity of the gill tissue from these populations and their structure at the cellular subcellular level could help determine this.

Although our focus here was on the 80+ genes involved in respiration directly, it is possible and highly likely that other genes participate in this process or may have been the primary target of adaptive evolution that affect downstream biological processes. For example, we previously showed that several heat shock protein (Hsp) genes are differentially regulated in response to heat in these strains (Narum et al. 2013). Heat shock proteins act as molecular chaperones in response to stress and notably, Hsp70 is known to chaperone the transcription factor “monocyte enhancement factor 2D” (MEF2D) into mitochondria to regulate the transcription of mitochondrial complex I genes (She et al*.* 2011). Furthermore, transcriptomic analysis of gene expression in these strains indicates substantially more differentially regulated genes in the desert strain and enrichment for genes involved in specific cellular processes (Narum and Campbell 2015). Finally, the fact that the expression pattern of the hybrid population did not match either parental strain suggests that nuclear-mitochondrial cross-talk and coadaptation may play a role.

From an applied standpoint for aquaculture, these gene expression patterns could be used for marker-assisted production of rainbow trout as a food source in facilities with high water temperatures. If this response is present in other species of Pacific salmon, it could be used to improve hatchery production. Indeed, a recent study in pink (*Oncorhynchus gorbuscha*) and sockeye (*Oncorhynchus nerka*) salmon did identify *COX6B1* as differentially regulated in response to heat (Jeffries et al*.* 2014). However, that study used microarrays to interrogate gene expression levels and it is not known whether the oligo on the array can detect differences between *COX6B1* and *COX6B2*, which would be important for a comparison to our results.

If this gene-expression profile is an adaptive response to increases in temperature in order to increase the efficiency of respiration and subsequent survival, it is potentially valuable for fisheries and conservation efforts. These patterns could be used to monitor the health of populations of fish that are exposed to higher temperature regimes as the climate continues to warm. Theoretically, populations that display these gene expression patterns may show some protection against the increased water temperatures and those that do not may be at risk.

In extreme cases where populations may be extirpated, fish with this response at elevated temperatures could be used to introduce adaptive gene complexes and increase species survival in those areas. The identification of the genetic variants that are responsible for these gene expression patterns would facilitate these efforts. An interesting avenue of research could also be the potential involvement of epigenetic factors such as DNA methylation or the involvement of microRNA or long nuclear RNA to regulate expression levels of the genes found here. Further studies that test for physiological adaptation among ecotypes (e.g., maximum thermal tolerance, cardiac performance [Eliason et al. 2011]) will also verify fitness advantages that may have evolved in desert populations over generations of exposure to warm water temperatures and low oxygen.

## Data Accessibility

The RNA-Seq data for this work are currently archived on the SRA in GenBank. The transcript counts and other data we extracted will be available on the DRYAD data repository.

## Supplementary Material

Supplementary tables S1 and S2 are available at *Genome Biology and Evolution* online (http://www.gbe.oxfordjournals.org/).

Supplementary Data
