# Supplementary material for: Differential Expression of Genes that Control Respiration Contribute to Thermal Adaptation in Redband Trout (Oncorhynchus mykiss gairdneri)
Source: Genome Biol Evol. 2015 May 4;7(6):1404–14. doi: 10.1093/gbe/evv078 (PMC4494065; doi:10.1093/gbe/evv078)
Supplement: Supplementary Data [file supp_7_6_1404__index.html]

Differential Expression of Genes that Control Respiration Contribute to Thermal Adaptation in Redband Trout (Oncorhynchus mykiss gairdneri) — Supplementary Data 

# Differential Expression of Genes that Control Respiration Contribute to Thermal Adaptation in Redband Trout (*Oncorhynchus mykiss gairdneri*)

## Supplementary Data

files

**Files in this Data Supplement:**

- Supplementary Data - xlsx file
- Supplementary Data - xlsx file
